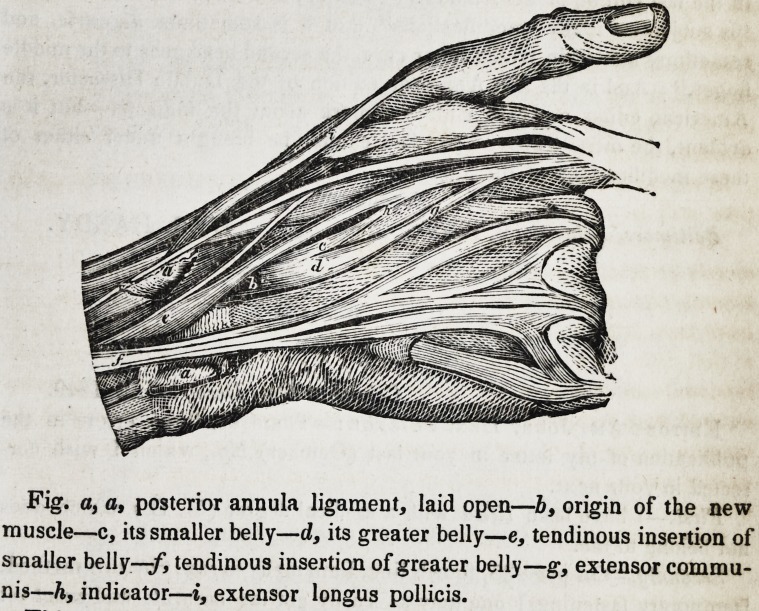# Miscellaneous Notices

**Published:** 1850-01

**Authors:** 


### Miscellaneous Notices.

*A Lump of Gold.*—While at Philadelphia last fall, we had the melancholy satisfaction of taking a small lump of gold into our hands, brought from California, by Lieutenant Beale. It weighed over seven pounds, seemed entirely free from foreign substances, and had every appearance of being quite pure. At the mint where it had been deposited for safe keeping, a heavy blade had clipped off a small protuberance nearly half an inch high, leaving a smooth, rich, and unbroken surface, of a little more than the same diameter, indicating, beyond all question, that it was "all gold that glittered," there, at least,

We confess to a slight glowing of the California fever, while the above was passing through our hands.

Several of our profession have gone thither with the intention of reversing their old habits, by *digging,* instead of depositing gold. Success attend them.—*Cazenovia Ed.*

---

*Arkansas Oil Stones.*—A few years ago, when the Arkansas oil stones first came into use with our profession, we deemed ourselves fortunate to obtain one, whose irregular and primitive form had been ground down on one side to a plane of an inch or two square. Now, they come to us perfected into almost every desirable shape. In the form of delicate, pearly blades, of every degree of thickness for finishing down stoppings between the teeth, and also in the shape of pencils of every character for polishing their grinding and buccal surfaces,—indeed, in almost every desirable form.

We hardly know where the tendency of manufacturing so stubborn a material will end, since human genius has attacked it, and we shall not be surprised to find some Arkansas man competing with the teeth manufacturers of our eastern cities, by producing incisores and cuspides, made of living crystals from nature's own laboratory! By the way, what capital instrument handles they would make. Think of a fine suit of them, octagon-shaped—no pearl would compare with them. What a pleasing combination of the useful, with the ornamental; each instrument would then contain a restorative quality within itself; and all lancets, especially, would have no excuse for being dull, so long as they could have an occasional rubbing against each other!

The improved Arkansas stones can be obtained at the principal dental depots in our large cities.

By calcining and reducing the stone to powder you obtain a very superior article for polishing the surfaces of gold stoppings, and also, for polishing plate. Drs. Cone and Austen made the suggestion to us while at Baltimore, last season.—*Cazenovia Ed.*

*Dry Cupping to relieve the Tooth-ache.*—In cases of tooth-ache, arising from inflammation of the dental periosteum, where the patient will submit to neither extraction or scarification of the gums, it may often be instantly relieved by applying one or two dry cups on the cheek over the part affected. It is true, cases like the above seldom occur, but the remedy is well worth remembering.—*Cazenovia Ed.*

*Accupuncturation in Facial Neuralgia.*—A few weeks since, a gentleman called upon us, who has been suffering for several years past with an obstinate facial neuralgia. The whole catalogue of remedies, such as leeches, blisters, liniments, ointments, strychnine, &c. &c., had been used in vain; he had experienced no relief, and his disease was steadily advancing. Having heard of my good fortune in curing a very protracted case of sciatica, by the use of the accupuncture needles, a year or two ago, he was anxious to have me insert them into the *supra-orbital nerve* of the left side of his face, the seat of his disease. On my inserting two of the needles, he expressed himself entirely free from pain, a relief he had not known for years. He remained with me a few days, during which, I repeated the introduction of the needles, when I dismissed him entirely cured. I received a letter from him but a day or two ago, in which he confirms, in the most enthusiatic terms, his complete recovery.

Practical Hints, No. 2.—Some time ago, we commenced a series of *practical hints,* intending to continue them in each number of the Journal; we have inadvertently failed to do so, but commence them again in the present issue.

*Working in Gold.*—Dentists who are not convenient to gold workers are generally obliged to manufacture their own plate; but whatever their advantages are in this respect, every mechanical dentist should have all of the facilities for working in gold, and acquire this art in all its branches, so far as it relates to his profession. There is as much difference between gold well prepared, and that which is not, as there is between good and bad foil.

A stubborn piece of gold of good degree of fineness, by dint of repeated meltings, and great care, is often worked into a very indifferent quality of plate, when, if a little precaution like the following had been used, it would have come from the crucible-like *flowing sovereigns, to melt gold filings, scraps, &c.*

Before putting the filings, scraps, &c, in the crucible, extract all of the iron and steel from it with a magnet. Put them into the crucible, then throw in a small quantity of sal æratus, and also a little nitre, then, into this, pour from one half to a tea-spoonful of nitric acid, according to the quantity of gold. Place the crucible on the fire, melt, and pour off. Your gold will be sure to work.

If you have an obstinate piece of gold, either granulate it, or roll it into thin strips and cut into minute pieces, then proceed as above.

*Muriate of Zinc*—to the matter of an ounce vial full, should have a place in every dentist's laboratory. For tinning iron, copper, or brass, and also, for furnishing a very neat, convenient, and rapid means of soft soldering, it is invaluable.

As much zinc as pure muriatic acid will dissolve, reduce one half with pure rain water, gives you the article desired. A slight wash of this liquid upon any of the above metals prepares them for a coating of solder, which may be effected by a slight blast of the blow-pipe.

*To make Plaster for Impressions set rapidly.*—In the former series of practical hints, we referred to various methods to effect this, such as by salt, alum, &c. &c; yet, we often practice the following: Put the plaster and water in a wedgewood mortar, and grind them thoroughly and rapidly together; this gives you a greatly improved and creamy *batter,* which will set almost immediately on being used.

*Shellac, or Dry Varnish*—may often be used to advantage, by giving a coat of it to a plaster impression, it may then be oiled, and on the plaster being cast into it the mould will part from the model without the least difficulty; the varnish prevents the oil from drying in. Varnishing the plaster models has long been practiced.

*Sheets of Wax to Prepare.*—When prepared in our mechanical department are too variously useful to specify. Take a smooth piece of fine board, say eight inches long, three or four inches broad, and a quarter of an inch thick; having a vessel of melted wax before you, first dip the board into the water, and then into the wax, removing it immediately, when you re-dip it until you have acquired the thickness desired. The whole may then be withdrawn from the wood in the form of a *case,* or *sheath,* and afterwards cut into any sized sheets required.

In this manner you may obtain sheets of wax from the thinness of writing paper to any desirable thickness.

*Wax to hold Gold Caps to their places.*—It is often exceedingly troublesome to make a gold cap keep its place during the early part of the operation of introducing foil into the cavity of the tooth. Several years ago, while Dr. Maynard, of Washington, was visiting us at our residence, he directed us, after perfectly drying out the cavity, to affix a small ring of wax around the under edge of the cap; then press it to its place over the exposed nerve, where, with a little care, it will remain until the foil has fully established it in its place. We have derived great benefit from the suggestion. Just before placing the cap over the exposed nerve, we generally give that organ the benefit of the smallest quantity of concentrated spirits of camphor. Our experience accredit advantages to the practice; inflammation seldom ensues after this precaution.

*Soap for Parting.*—It is useful for all green moulds, and is particularly convenient when one is in haste. Sculptors always use this parting for their first, or *waste* mould. Common soap will do, but any of the fancy soaps dissolved in water, to the consistence of cream, are better. Apply with a fine brush. The smaller the quantity you use the better, providing you are particular to coat the *whole surface.* The only precaution necessary, is to part the cast from the mould immediately after the plaster is thoroughly set.

*Mix your Borax with Soft Water.*—Many an excellent dental fixture, and many a good temper has been spoiled in the bargain, by grinding borax for soldering, with *hard,* instead of soft water, especially by those living in calcareous locations.

We regard the manufacture of *glass* as one of the useful and elegant arts, but sadly out of place when it steals into the laboratory of the dentist, and arrests the progress of soldering, by interposing a silicious barrier between opposing parts of his work. Had mechanical dentists flourished before Pliny's time, the river Belus would never have been surprised at finding vitrious coated stones on her shore!

*Gold Pivots.*—We have usually inserted in the following manner: The pivot having been previously well fitted to the hollow screw within the fang, is mounted upon the tooth to be inserted; with a sharp instrument the whole of its exposed surface is cut up into numerous small barbs, opening downwards, then with a watch-spring saw, the pivot is split about one half its length; the two branches thus made, are slightly separated, yet so as to spring together by pressure. The edges of the top of the pivot is trimmed down with a file, so as to admit of its entering the cylinder, when it is forced to its place.

When thus mounted, we have never been troubled by teeth coming out, or being displaced. The *action* of the pivot is self-evident.

*Splitting the rivets of Plate Teeth,* is every way preferable to the usual method of heading them down with a hammer. Teeth are often cracked at the out-set, or are so strained by the heading process that they crumble away, and part from the rivets on being heated for soldering.

For linings, we use plate, No. 26. After counter-sinking, and fitting the lining to the tooth in hand, we file down the rivets even with the gold, then, with a small chisel-shaped instrument, a little broader than the rivets, we press down upon them until we have split, or broken up its surface into numerous points, at each effort, the outer portions of the rivets are thrown back upon the linings, this binds it effectually to its place. But there are yet greater advantages arising from this method. By splitting the rivets as above, the surface of the platina rivets to be covered by the solder is increased by manifold, while from the fact of their being absolutely beneath the surface of the lining they are not exposed by finishing.

*Drilling Dental Blocks.*—Those of our profession who reside in large cities where they can command the services of a lapidary, possess advantages over us, in less favored locations, which are decidedly unquestionable; yet, our deficiencies, in this respect, may nevertheless be greatly overcome by patience and ingenuity.

It has been our misfortune, on several occasions, to break pieces of dental blocks, oftentimes, too, after soldering sets of single gum teeth, we have discovered the *gum* part of some one of them cracked off; yet in neither instance have we despaired of correcting the evil and saving the pieces at the same time, A common drill, made of good steel, and of hardest temper, driven with a bow, or the lathe, if it be occasionally replenished with oil to facilitate its cutting, and preserve its temper, and also, if it be re-tempered, and re-sharpened a few times, will soon pass through the hardest dental substitute that ever was vitrified by heat. We confess the lapidary is enabled to work much more rapidly, but when we contemplate him some three hundred miles distant, we can easily reconcile ourselves to the extra hour or so, required to accomplish the same thing. A piece of artificial gum of a single tooth can be perforated in a comparatively short time, and then nicely fitted to its place on the plate, by a small gold rivet. A few days ago, we saw a full upper set of gum teeth, a section of the gum of which we treated in this way six years ago; a a small hair line only, indicated the fracture, the gold rivet was too high to be perceptible. Speaking of highly tempered steel drills reminds us of several methods we have practiced.

*To Temper Steel Drills*—so as to give them the quality of toughness with exceeding hardness. While in Baltimore last season, Dr. Cone suggested to us to heat the drill to redness, and then plunge it into red sealing wax; this we find preferable to using either water or oil; but we have found nothing equal to plunging them, while of a cherry red heat, into a mixture composed of one ounce of calomel, mixed with a pint of pure water.

It is often desirable to harden the point simply, of drills, excavators, blades of instruments, &c., leaving the rest of them in their untempered state. This is effected by placing your dish of liquid on a line below, and immediately by the side of the lighted lamp, then holding the instrument so that its point is but two or three lines above the fluid mixture, you direct the smallest blaze of a blow-pipe upon its extreme point; the instant it acquires cherry redness, drop it into the bath below, and you have accomplished your object. In this manner a breadth of a quarter of a line of a delicate instrument may be made of the greatest degree of hardness, while the rest remains wholly unchanged.—*Cazenovia Ed.*

*The Diploma of the Baltimore College of Dental Surgery.*—We have received from England several applications relative to the purchase of a diploma from the above institution. We answer the writers, by stating that when they have gone through the curiculum of study prescribed by its charter, and submitted to the usual examination, they will be entitled to a diploma. We have also been informed that several persons are using the title who possess no diploma from this Institution. We shall consequently furnish our correspondent, Dr. James Robinson, of London, with the names of all to whom it has been granted in Great Britain.—*Balt. Ed.*

*Dental Medicine.*—We commence in the Library part of the present number of the Journal, the publication of an original work on Dental Medicine, by Professor T. E. Bond, M. D., which, we have no doubt, will be gladly received by our subscribers. Such a work has been long needed, and we know of no one better qualified to prepare such a book than Dr. Bond. As a chaste and classical writer he has few equals, and we have no doubt he will do ample justice to the task he has undertaken. The work, however, requires no commendation from us; it will speak for itself. As nearly as we can ascertain, it will be comprised in from four to five hundred pages.—*Balt. Ed.*

*Professional Jealousy in England.*—Dr. James Robinson, our European correspondent, promises in a communication to us, to forward for publication in the next Journal, the particulars, with copies of correspondence &c., relating to his appointment as Surgeon Dentist to Prince Albert. This extraordinary affair, so far as we understand it, has excited much interest among the profession generally, and the details will, we understand, contain a serious charge against a brother, professional, in England.—*Balt. Ed.*

*To Correspondents.*—We have received several very interesting communications for publication, which, for want of room, we are compelled to omit in our present number. They shall appear in our next.—*Balt. Ed.*

*Researches on the Development and Structure of the Human Teeth, by* ALEXANDER NASMYTH.—We have only space in this number to announce the publication of the above named work. It has been long looked for by the profession, and its appearance will, we have no doubt, be cordially welcomed. We shall notice it at length in our next.

*Balt. Ed.*

*An Imposter.*—Some three or four weeks since, we received a letter from Dr. Morgan, of Nashville, Tenn., accompanied by an advertisement of a man, calling himself a dentist, by the name of H. K. Neigh, in which he says he is a graduate in medicine, and of two colleges of dental surgery. He also refers for professional ability, &c., to Drs. E. Parmly, of New York; Maynard, Washington City; Hullihen, Wheeling, Va.; Townsend, Philadelphia, and C. A. Harris, Baltimore.

It is scarcely necessary to say that Mr. Neigh is not a graduate of the Baltimore College of Dental Surgery, that we never heard of him before, and that consequently he has used our name without our knowledge, or permission, as we doubt not, he has the names of the other gentlemen above referred to.

Within the last day or two, we received a letter from Dr. Charles W. Snow, of Eufaula, Alabama, stating, that "a stranger, calling himself H. Nye, M. D., came to" that "place some ten days" before, "stating that he was a dentist, had been engaged in the profession some ten years, studied with" us "in Baltimore, graduated at the Baltimore Dental College, had done a great deal of work for us in our office, and exhibiting certificates from" us "and Dr. Eleazar Parmly, of New York." From the foregoing representation, Dr. S., who is a dentist, was induced to take him into copartnership, but soon after, an Augusta paper was handed to him by a friend, "containing an advertisement, copied from the

Jacksonian, published at Holly Springs, Miss., stating that a man, calling himself H. K. Neigh, came there from Bolivar, Tenn., in October last, passed as a dentist, left in about two weeks with a buggy and two horses, hired of Messrs. Stone & Cook, to go a few miles into the country to do some work; the next thing heard from him was in Tuscumbia, Alabama; he had sold the horses and buggy, and left for the east—some gentlemen with him from Tuscumbia, finding him changing his name, became suspicious, and compelled him to refund the money."

The paper containing the above statement having been shown to Dr. Nye, and the citizens of Eufaula suspecting that he was the individual referred to in it, he proposed to clear himself by writing to us, and the following is the letter which we received:

*Eufaula, February 7th,* 1850.

Chapin A. Harris, M. D., D. D. S.:

*Dear Sir:*—I arrived in this place a few days since, and entered into copartnership with Dr. C. W. Snow; but scarcely had I done so, before some newspapers were received here, containing an advertisement of a dentist by the name of H. K. Neigh, who has been in different places swindling the people in various ways. He is represented as being a fine looking man, and as wearing spectacles. Now as *I* wear glasses, the people here have come to the conclusion, that as my name is Nye, so nearly corresponding to the name of this scamp, that I am none other than the veritable scoundrel himself. Now, my object in writing to you at present, is to have you write back a letter to me, saying to the people here whether my name is, or is not, H. Nye, M. D., and whether I did, or did not, study with you, and graduate in the Baltimore Dental College.

Now, I wish you to write out a certificate of my name, character, and standing in Baltimore, together with the length of time I have been engaged in the dental profession, and sign it yourself, and get Drs. Bond, Blanden, McCook, and George Austen to put their names to it, and send it to DRS. SNOW & NYE,

*Eufaula, Barbour Co., Ala.*

P. S.—Please write with all possible haste on the receipt of this, as it is very unpleasant to lay under such suspicions.

I am, sir, very respectfully, your obedient servant,

DR. H. NYE.

Still further, please state whether I was, or was not, in Baltimore from first of September last, until I left for the South, and also the *time* I left for the South. Dr. H. N.

In reply to the above letter, we can only say, that Dr. H. Nye, is not a graduate of the Baltimore College of Dental Surgery, that no such man ever studied in our office, or received from us a certificate of professional ability.—*Balt. Ed.*

*Highly Creditable.*—At the request of Mr. Evans, we copy the following from the Dental News Letter. The result of Mr. E's experiments with amalgam is precisely what we feared it would be, and the course which he has taken in this matter reflects upon him the highest credit.

*Balt. Ed.*

Messrs. Jones, White & Co.

*Gentlemen:* In my last communication, I gave the result of my experiments with the amalgam of tin and cadmium up to that date. The result had not been as satisfactory as it seemed to promise in the commencement.

The deep yellow color, I mentioned as having observed in some cases beneath the filling, upon removing it, caused me to fear it would not be durable. Since which time, I have examined some of the early cases in which I employed this filling; some have entirely failed, while others are apparently doing well. In all the cases, the filling upon the surface appears to retain its color. Finding it to differ so much in different cases, I am induced to regard it as at least an uncertain article. I do not feel satisfied to use it, even as an expedient, under such circumstances; having no confidence myself in its durability, I do not even feel justified in recommending its use to the profession.

In regard to its merit, as compared with the various other amalgams, time will be its best test.

My experiments some years ago, with this preparation, were not sufficiently protracted to enable me to discover the phenomenon that I have recently observed; nor were my means of observation so extended, as they have been latterly, from the fact, that my experiments were discontinued at that time. I was induced to believe, at a very early stage of my professional career, that all preparations for filling teeth in which mercury entered as a component part, were objectionable, which I believe is the opinion of many of our American dentists; indeed, there has always existed more or less prejudice in the public mind, against mercurial preparations.

It was not until I had been in Europe some time, and had found that the practice of filling teeth with amalgams was so universal, that I was induced to resume my experiments with the preparation. Believing it to be better than those amalgams in general use, I hoped it might prove of some utility to the profession, and also, that humanity at large would be benefited by it.

THOS. W. EVANS.

*Paris, December* 11, 1849.

*Letter from* Dr. W. R. Handy, to the Baltimore Editor: *Dear Doctor:* I place at your disposal an extract from my note book, in which are recorded things of an anatomical interest, that may be observed in the dissections carried on by the students, during the regular course of in struction in the Baltimore Dental College.

The extract refers to a muscle, entirely new, so far as my dissections, and the examination of such standard anatomical works as in my possession are entitled to a decision of the matter. Accompanying this, I send you a drawing of the muscle, by Mr. Woodward, a professed artist, and one of our own students, whose faithfulness of exhibition not only declares his competency for the task, but further demands my feeble tribute of approbation for his professional artistic talents which so justly merit encouragement.

Fig. *a, a,* posterior annula ligament, laid open—*b,* origin of the new muscle—c, its smaller belly—*d,* its greater belly—*e,* tendinous insertion of smaller belly—*f,* tendinous insertion of greater belly—*g,* extensor communis—*h,* indicator—*i,* extensor longus pollicis.

This muscle was seen during our dissections, in the month of January last, on both hands of a colored woman, and which, from its attachments and function we have thought proper, as not unappropriate, to call *the extensor accessorius indicis.*

It was seen to *arise* upon the right hand by a delicate tendinous membrane, from the radio-carpal articulation behind the posterior annular ligament, and in the same groove with, and posterior to, the tendons of the extensor communis, and indicator—becoming fleshy, it soon divides into two bellies of different size—the smaller belly connects with the tendon of the *indicator* near the base of the metacarpal bone—the larger, about two inches in length, connects also with the tendon of the indicator, but near the articulation of the metacarpal bone, with the first phalanx of the forefinger, by a slender tendon as represented in the drawing. On the left hand, the muscle had a similar *origin,* but had only one fleshy belly, which ended in a like tendon, having the same *insertion* or attachment with the larger belly upon the right. The size of the muscle in either hand was nearly equal to that of the plantaris in the leg.

*Function.*—Its use seems evidently to assist the indicator, in the extension of the fore-finger.

The extensor communis on each hand sent its usual tendon to both index fingers, and united in the usual way with the indicator.

*Remarks.*—The drawing represents the posterior annular ligament laid open and the extensor communis turned to one side. Dr. Horner, in the last edition of his Anatomy, remarks, in a note, that the indicator "is subject to many modifications" that it is sometimes *digastric,* and sometimes *double,* in which latter case, the second head goes to the middle finger." And in the first American edition of the Dublin Dissector, the American editor makes a similar remark about the *indicator*—but it is evident, the muscle above described cannot be brought under either of these modifications of the indicator.

Yours, truly,

*Baltimore, February* 2, 1850. W. R. HANDY.

---

*Columbus Ga., December* 12, 1849.

EDITORS AM. JOUR. DEN. SCIENCE:—There are three errors in the publication of my letter in your last (October) No., which I wish corrected in your next.

*First.*—I have been *titled* with a medical degree (M. D.) which does not belong to me.

*Secondly.*—On page 35, fourth line, I am made to say "by this method, [temporary fastenings] one may as easily get the *accurate* and *exact* set of the clasps as *by the mouth.*"

The word AS was not written; and you will see it completely reverses my meaning.

*Thirdly.*—On the same page, the last sentence reads "file it—[the end of the bow] to make a *point.*" I wrote *joint;* which every one, who would solder two pieces together, should make it a "point" to make.

Will the *readers* of the "Journal" also please mark these corrections on their pages?

Very respectfully,

C. T. CUSHMAN.

## Figures and Tables

**Figure f1:**